# DECLINE IN EXERCISE IN OLD AGE: LONGITUDINAL EVIDENCE OF DISTANCE TO DEATH AND THE ROLE OF SELF-PERCEPTIONS OF AGING

**DOI:** 10.1093/geroni/igae098.3477

**Published:** 2024-12-31

**Authors:** Zheyuan Zhang, Wan Wen, Shannon Mejia

**Affiliations:** Beijing Normal University, Beijing, Beijing, China (People’s Republic); University of Illinois Urbana-Champaign, Champaign, Illinois, United States; University of Illinois Urbana-Champaign, Urbana, Illinois, United States

## Abstract

As individuals age, they are more likely to face health issues and physical mobility constraints associated with mortality, resulting in a decrease in engagement in physical activities during their later life stages. This decrease in activity can adversely impact their longevity and well-being. Self-perceptions of aging (SPA) constitute a crucial psychological resource for older adults. More positive SPAs have a protective effect against health decline and encourage the adoption of healthier behaviors. However, the impact of SPA on health behaviors towards the end of life, especially in moderating the relationship between the proximity to death and exercise engagement, remains insufficiently explored. This study used 10-year longitudinal data from deceased participants of the Chinese Longitudinal Healthy Longevity Survey (CLHLS) (N=9,778, age range:62~117, 59% female). After excluding observations with missing data, a total of 12,027 observations were analyzed. Multilevel models showed that as older adults approached the end of life, their likelihood of engaging in physical activity decreased. However, positive SPA can mitigate this decline. These insights underscore the importance of SPA in health promotion interventions aimed at enhancing physical activity among older adults, offering strategies to increase exercise participation in their later years.

